# A* Fomitopsis pinicola Jeseng* Formulation Has an Antiobesity Effect and Protects against Hepatic Steatosis in Mice with High-Fat Diet-Induced Obesity

**DOI:** 10.1155/2016/7312472

**Published:** 2016-04-20

**Authors:** Hoe-Yune Jung, Yosep Ji, Na-Ri Kim, Do-Young Kim, Kyong-Tai Kim, Bo-Hwa Choi

**Affiliations:** ^1^Pohang Center for Evaluation of Biomaterials, Pohang Technopark, Pohang 37668, Republic of Korea; ^2^Department of Life Science, Division of Integrative Biosciences and Biotechnology, POSTECH, Pohang 37673, Republic of Korea; ^3^R&D Center, NovMetaPharma Co., Ltd., Pohang 37668, Republic of Korea; ^4^School of Life Science, Handong Global University, Pohang 37554, Republic of Korea

## Abstract

This study investigated the antiobesity effect of an extract of the Fomitopsis pinicola Jeseng-containing formulation (FAVA), which is a combination of four natural components:* Fomitopsis pinicola Jeseng*;* Acanthopanax senticosus*;* Viscum album coloratum*; and* Allium tuberosum*. High-fat diet- (HFD-) fed male C57BL/6J mice were treated with FAVA (200 mg/kg/day) for 12 weeks to monitor the antiobesity effect and amelioration of nonalcoholic fatty liver diseases (NAFLD). Body and white adipose tissue (WAT) weights were reduced in FAVA-treated mice, and a histological examination showed an amelioration of fatty liver in FAVA-treated mice without decreasing food consumption. Additionally, FAVA reduced serum lipid profiles, leptin, and insulin levels compared with the HFD control group. The FAVA extract suppressed lipogenic mRNA expression levels from WAT concomitantly with the cholesterol biosynthesis level in the liver. These results demonstrate the inhibitory effects of FAVA on obesity and NAFLD in the diet-induced obese (DIO) mouse model. Therefore, FAVA may be an effective therapeutic candidate for treating obesity and fatty liver caused by a high-fat diet.

## 1. Introduction

There is increasing consensus that obesity may be the main cause of various metabolic disorders. Obesity is caused by the combined effects of excess energy intake and reduced energy expenditure. It is one of the fastest growing disorders worldwide and is associated with various clinical symptoms in developed countries [1], such as hyperlipidemia, insulin resistance, and nonalcoholic fatty liver diseases (NAFLD) [2]. It is well known that excessive fat consumption is implicated in the development of obesity in mice [3], and long-term feeding with a high-fat diet (HFD) can induce obesity together with hyperlipidemia, insulin resistance, and NAFLD [4]. Hyperlipidemia is associated with high levels of lipids and lipoproteins in the blood and causes atherosclerosis and acute pancreatitis [5]. Although NAFLD is the second leading cause of death in the general population [6, 7], there is no pharmacological agent known to reverse NAFLD. Recently, effective medical interventions have been focused on the modification of risk factors, such as diet and weight reduction [8].

Adipose tissue, an important repository for energy storage, regulates energy homeostasis. Adipogenesis, a differentiation process of adipocytes, involves changes in gene expression and cellular morphology. Adipocyte hypertrophy results from an excessive accumulation of lipids from the intake of inordinate energy sources such as HFD. During adipogenesis, peroxisome proliferator-activated receptor-*γ* (PPAR-*γ*) and CCAAT/enhancer-binding protein-*α* (C/EBP-*α*) play key roles as major transcriptional factors [9]. Expression of PPAR-*γ*, a transcription factor of the nuclear-receptor superfamily, and C/EBP-*α*, a member of the C/EBP family of basic leucine zipper class of transcription factors, increases during 3T3-L1 cell differentiation [10]. Lipin is also a central regulator of adipose tissue development. Mammalian lipin proteins have been shown to control gene expression and to enzymatically convert phosphatidate into diacylglycerol, an essential precursor in triacylglycerol and phospholipid synthesis [11]. Previous studies have established that lipin-1 is required at an early step in adipocyte differentiation for the induction of the adipogenic gene transcription program, including the key regulator PPAR-*γ* [12].

Acetoacetyl-CoA synthetase (AACS) regulation is related to cholesterol and lipid homeostasis [13]. Sterol response element binding protein-2 (SREBP-2) may play an essential role in the transcriptional regulation of AACS. SREBP-2 is a leucine zipper transcription factor that controls a rate-limiting enzyme in cholesterol synthesis, HMG-CoA reductase (HMGCR), when the factor binds sterol response element [14, 15].

The effects of* Acanthopanax senticosus*,* Allium tuberosum,* and* Viscum album coloratum* have been studied for inhibition of fatty acid synthase and prevention of obesity, as well as reducing hepatic steatosis [16–18].* Fomitopsis pinicola Jeseng* has been reported for antihyperglycemic effect in diabetic rats [19]. In addition, it has been reported that *β*-glucan-rich extract, a major component of* Fomitopsis pinicola Jeseng*, effectively reduces adiposity [19, 20]. However, to our knowledge, no reports are available on the effect of* Fomitopsis pinicola Jeseng* on obesity.

In this study, we investigated the effect of FAVA in a high-fat diet-induced mouse model. The FAVA is a combination of herbal extracts (i.e.,* Fomitopsis pinicola Jeseng*,* Acanthopanax senticosus*,* Allium tuberosum,* and* Viscum album coloratum*) at a ratio of 5 : 3 : 1 : 1. This study investigated the effect of a mixture containing dietary components on metabolic disorders including obesity, hyperlipidemia, and NAFLD using a high-fat diet-induced obesity mouse model and the molecular mechanism level of adipogenesis and cholesterol biosynthesis. Our results indicate the great potential of FAVA as a potential metabolic regulator of adipogenesis and cholesterol biosynthesis and as a potential therapeutic agent for preventing or treating obesity and NAFLD.

## 2. Materials and Methods

### 2.1. Preparation of FAVA

The oriental, medicinal, and herbal mixture used in this experiment (FAVA) was prepared as described previously [21–23]. Briefly,* Acanthopanax senticosus* and* Allium tuberosum* were extracted with 80% methanol while water extracted* Fomitopsis pinicola Jeseng* and* Viscum album coloratum* were purchased from commercial vendor (Mistle Biotech Co., Ltd., Korea). Each extraction of FAVA was resuspended in distilled water (DW) in a ratio of 50%, 30%, 10%, and 10%, respectively, and prepared in appropriate diluent for further* in vivo* study.

### 2.2. Animals and Diets

Lean, male C57BL/6J mice (7 weeks old) were purchased from Charles River Laboratories Japan, Inc. (Yokohama, Japan). All animal experiments were approved by the Ethics Review Committee of the Pohang Center for the Evaluation of Biomaterials, Republic of Korea. All mice were housed for 1 week under a 12/12-h light/dark cycle in a temperature- (22 ± 1°C) and humidity- (55 ± 5%) controlled room and fed standard laboratory chow and water* ad libitum* while FAVA, orlistat, and saline supplementation were performed using oral gavage once a day. To induce obesity, the mice were fed a HFD (Rodent Diet D12492, Research Diet, New Brunswick, NJ, USA) consisting of 60% kcal fat. Control mice were fed a low-fat chow diet (Rodent Diet D12450B, Research Diet, New Brunswick, NJ, USA) consisting of 10% kcal fat. Experimental mice were given FAVA or orlistat as a positive control (Chongqing Zein Pharmaceutical Co., Ltd., Chongqing, China). The mice were randomly divided into four groups (*n* = 8 per group) that were fed a low-fat chow diet (CHOW), a high-fat diet (HFD), HFD plus FAVA (200 mg/kg/day), or HFD plus orlistat (60 mg/kg/day). Animals were fed via oral feeding needles for 12 weeks, and the CHOW and HFD group received an equivalent volume of saline. Body weight was measured once a week, and food intake was measured three times per week during the course of the study. At the conclusion of the* in vivo* experiment, the mice were sacrificed by cervical vertebral dislocation, and the epididymal, mesenteric, and subcutaneous fat pads and liver were collected and weighed. The epididymal fat pad samples were stored at −80°C until analysis.

### 2.3. Serum Analysis

Serum was collected by cardiac puncture, stored for 20 minutes at room temperature for coagulation, and then separated by centrifugation at 2,000 ×g for 20 minutes. The serum was stored at −70°C until analysis. The levels of triglycerides, total cholesterol, high-density lipoprotein (HDL) cholesterol, low-density lipoprotein (LDL) cholesterol, glucose, alanine transaminase (ALT), aspartate transaminase (AST), BUN, and creatinine in serum were measured by using an automated biochemical analyzer (BS-390, Mindray Bio-Medical Electronics Co., Ltd., China).

### 2.4. Measurement of Leptin and Insulin

The leptin and insulin concentrations in serum were determined by a mouse enzyme-linked immunosorbent assay (ELISA) kit (Morinaga Institute of Biological Science, Yokohama, Japan). The preparation of serum samples is described above.

### 2.5. Abdominal Computed Tomography Analysis

Experiments of micro-computed tomography (micro-CT) were performed with an animal positron emission tomography (PET)/CT/single photon emission computed tomography (SPECT) system (Inveon, Siemens, USA) prior to the sacrifice of animals under 1.5–2% isoflurane in O_2_ anesthesia. Computed tomography pictures were further analyzed using Siemens Inveon software to calculate the three-dimensional volume of the fat mass between lumbar vertebrae one to five.

### 2.6. Liver Histology

Liver tissues were immediately isolated after sacrifice. For hematoxylin and eosin (H&E) staining, the tissues were fixed in 10% formalin, processed, and embedded in paraffin prior to sectioning (10 *μ*m) and staining. The liver samples of 3 mice from each group (CHOW, HFD, HFD + FAVA, and HFD + ORLISTAT) were measured. Briefly, the following criteria were used for scoring hepatic steatosis: grade 0 (no fatty liver) and grade 1 (mild fatty liver), if hepatocytes occupied <33% of the hepatic parenchyma [24].

### 2.7. RNA Preparation and Real-Time PCR

Total RNA was extracted by ReliaPrep RNA Tissue Miniprep System (Promega) according to the manufacturer's instructions. RNA integrity was assessed by an automated microfluidics-based system (Bioanalyzer 2100, Agilent, Palo Alto, CA, USA). First-strand cDNA was synthesized with the iScript cDNA Synthesis Kit (Bio-Rad, Hercules, CA, USA), and real-time PCR was performed using an iCycler iQ Real-Time Detection System (Bio-Rad). PCR reactions were conducted with iQ SYBR Green Supermix (Bio-Rad). Real-time PCR analysis was performed using an iCycler iQ Real-Time Detection System (Bio-Rad). Amplification of real-time PCR was performed according to the protocols of Jung et al. [25]. The reaction was performed at 95°C for 3 min, followed by 39 cycles of amplification (95°C for 10 s, 58°C for 10 s, and 72°C for 30 s). A melting curve was produced to confirm a single gene-specific peak and detect primer/dimer formation by heating the samples from 65 to 95°C in 0.5°C increments with a dwell time at each temperature of 10 s, while continuously monitoring fluorescence. The mRNA levels of specific genes were normalized to those of *β*-actin. The primers used are listed in Table 1.

### 2.8. Statistical Analyses

The data (mean ± SE) were analyzed using GraphPad Prism (version 5.04, GraphPad Software, USA). Unpaired two-tailed Student's *t*-tests were used to evaluate differences between means as indicated and *p* values < 0.05 were considered significant.

## 3. Results

### 3.1. Effects of FAVA on Body Weight, Dietary Intake, and Fat Mass in White Adipose Tissue in HFD-Fed Mice

**Figure 1:**
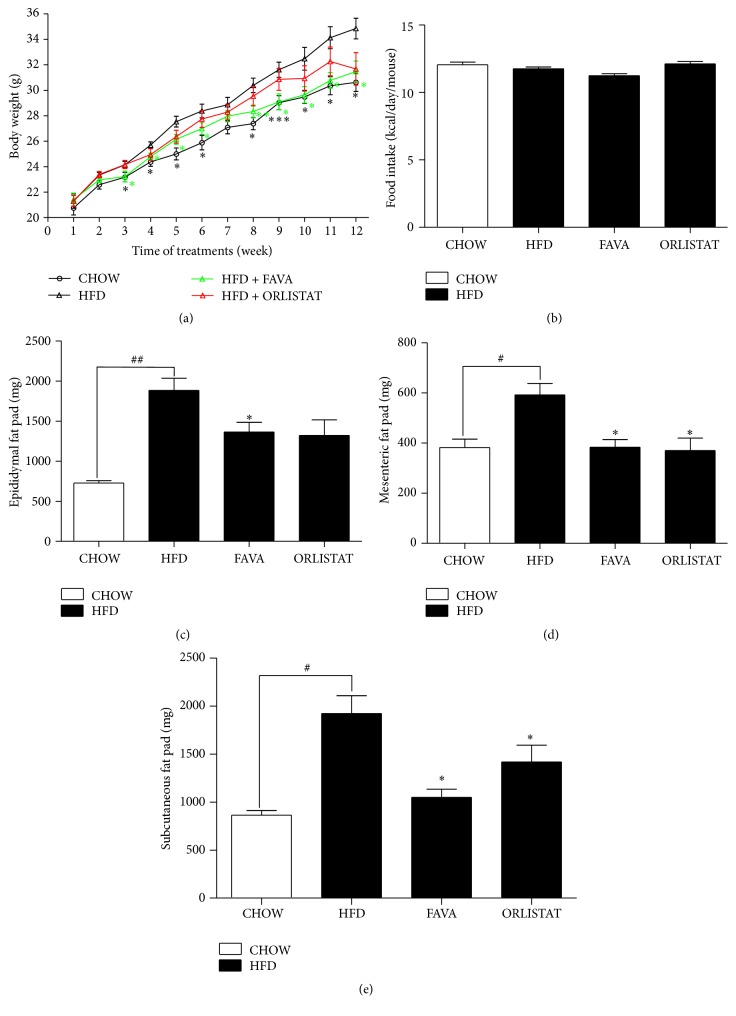
Effect of FAVA on body weight, food intake, and white fat pad in mice fed a high-fat diet for 12 weeks. (a) Changes in body weight gain at each treatment period are shown: (circle) CHOW: chow diet; (black triangle) HFD: high-fat diet; (red triangle) HFD + ORLISTAT: high-fat diet plus orlistat 60 mg/kg; and (green triangle) HFD + FAVA: high-fat diet plus FAVA 200 mg/kg. (b) Average food intake expressed as kcal/mouse/day. ((c)–(e)) Epididymal fat pad (c), mesenteric fat pad (d), and subcutaneous fat pad (e) weights expressed. The values represent the mean ± standard error of mean (SEM) (^#^ *p* < 0.05 and ^##^ *p* < 0.005 versus the CHOW group; ^*∗*^ *p* < 0.05, ^*∗∗*^ *p* < 0.005, and ^*∗∗∗*^ *p* < 0.0005 versus the HFD group, *n* = 8 per group).

The effects of FAVA on body weights are shown in Figure 1(a). During the 12-week experiment, body weight was measured weekly, and food intake was measured every other day. After 9 weeks, the body weight of the mice in the HFD group was significantly higher than that of the mice in the CHOW group (*p* < 0.0005). The FAVA-treated group showed a significant decrease in body weight compared with the HFD group. At the end of the experiment, the body weight of the mice fed FAVA was 9.7 ± 2.0% lower (*p* < 0.05) than that of the mice in the HFD group, whereas HF diet plus orlistat-fed mice weighed almost the same as the mice that were fed FAVA (Figure 1(a)). These effects of FAVA on body weight were not due to decreased food intake, because the amount of kcal consumed per mouse over a 24-h period remained unchanged (Figure 1(b)). These data indicate that FAVA might have antiobesity effects* in vivo*, without affecting food intake. To investigate whether body weight loss was caused by decreased adiposity, the animals were sacrificed, and the epididymal fat pad, the mesenteric fat pad, and the subcutaneous fat pad were dissected and weighed. FAVA supplementation significantly suppressed the increase of fat mass in all white adipose tissues, including mesenteric, subcutaneous, and epididymal adipose tissue (Figures 1(c)–1(e)).

### 3.2. Effect of FAVA on Adiposity in HFD-Fed Mice

**Figure 2:**
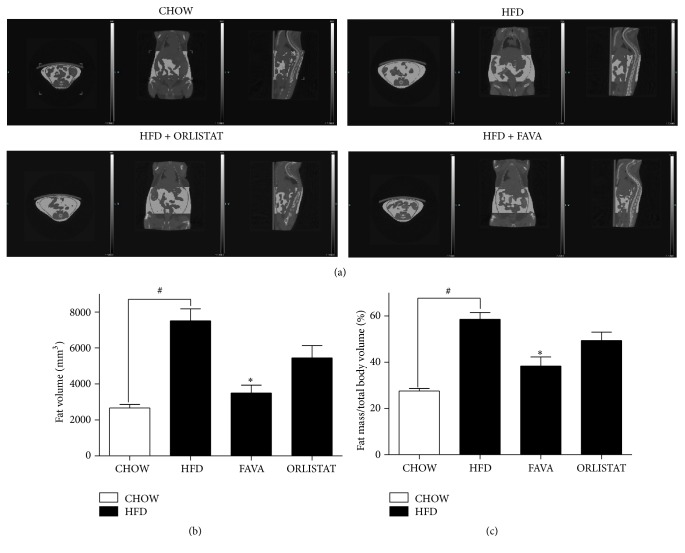
Effect of FAVA on high-fat diet-induced adiposity. (a) Micro-computed tomography (micro-CT) pictures were analyzed using Siemens Inveon software to calculate the three-dimensional volume of the fat mass between vertebrae number one to five of mice fed a chow diet (CHOW), high-fat diet (HFD), HFD with 60 mg/kg/day orlistat (ORLISTAT), or HFD with 200 mg/kg/day FAVA (FAVA). (b) Fat volumes (mm^3^) in mice are shown. (c) Fat pad mass expressed as percentage of total body weight. The values represent the mean ± SEM (^#^ *p* < 0.05 versus the CHOW group; ^*∗*^ *p* < 0.05 versus the HFD group, *n* = 3).

We performed micro-CT imaging to assess the effect of FAVA on adiposity. CT imaging showed a significant reduction in body fat profiles with FAVA treatment (Figure 2(a)). There was a significant reduction in fat volume (Figure 2(b)) and total body fat percentage in FAVA-fed groups compared with the HFD group (Figures 2(b) and 2(c)).

### 3.3. Effects of FAVA on Serum Insulin, Leptin, and Lipid Profiles in the Serum of HFD-Fed Mice

**Figure 3:**
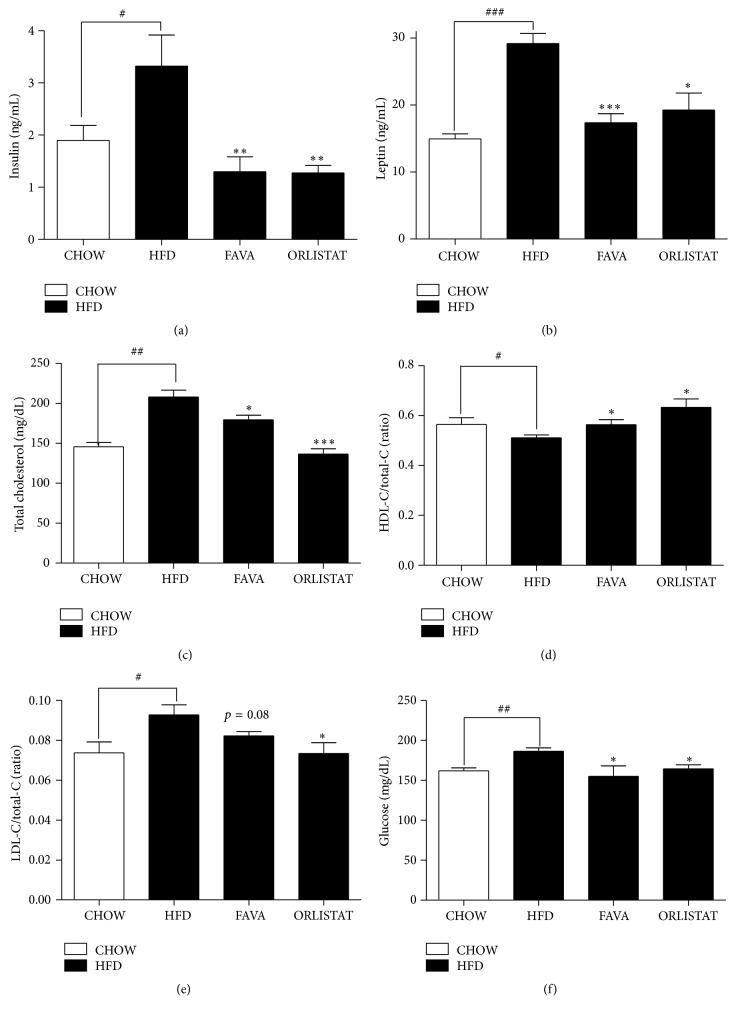
Effect of FAVA on serum insulin, leptin, and lipid profiles in mice fed a high-fat diet. Changes in insulin (a), leptin (b), total cholesterol (c), the ratio of HDL cholesterol/total cholesterol (d), and the ratio of LDL cholesterol/total cholesterol (e) of the mice were measured. The values represent the mean ± SEM (^#^ *p* < 0.05, ^##^ *p* < 0.005, and ^###^ *p* < 0.0005 versus the CHOW group; ^*∗*^ *p* < 0.05, ^*∗∗*^ *p* < 0.005, and ^*∗∗∗*^ *p* < 0.0005 versus the HFD group, *n* = 5~7 per group).

The changes in the blood plasma parameters are shown in Figures 3(a)–3(e). As shown in Figures 3(a) and 3(b), HFD-induced obese mice showed significantly higher levels of serum insulin and leptin, whereas the FAVA group showed significantly decreased levels of serum insulin and leptin by 60.9 ± 8.1% and 40.4 ± 3.0%, respectively. Concomitant reductions of serum insulin and leptin levels were monitored in the orlistat group in a similar manner. Additionally, the FAVA group showed lower levels of serum total cholesterol and the ratio of LDL cholesterol/total cholesterol than those of the HFD group.

### 3.4. Effects of FAVA on mRNA Levels of Transcriptional Factors in Epididymal Fat Pad

**Figure 4:**
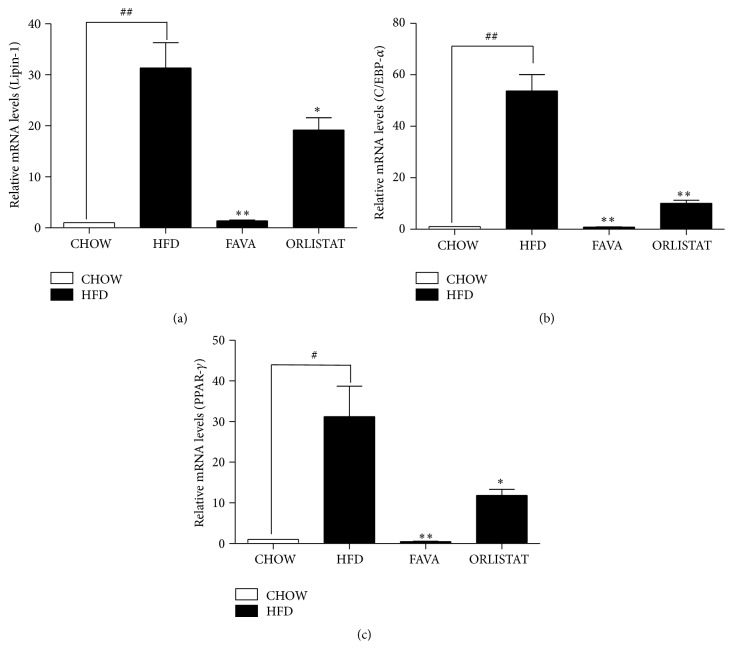
mRNA expressions of transcription factors in the epididymal fat pad of animals treated with HFD or HFD + ORLISTAT (high-fat diet plus orlistat 60 mg/kg) or FAVA (high-fat diet plus FAVA 200 mg/kg) or chow as quantified by real-time PCR. The graphs represent mRNA expression of transcription factors Lipin-1 (a), ACC and C/EBP-*α* (b), and PPAR-*γ*. The data represent the mean ± SEM (^#^ *p* < 0.05 and ^##^ *p* < 0.005 versus the CHOW group; ^*∗*^ *p* < 0.05 and ^*∗∗*^ *p* < 0.005 versus the HFD group, *n* = 5).

Because FAVA extract reduced fat mass in all white adipose tissues and serum insulin levels (Figures 1 and 3), we evaluated the effect of FAVA on the expression of various adipogenic and lipogenic genes [26]. PPAR*γ* and C/EBP*α* are known to have roles in insulin sensitivity, lipogenesis, and lipolysis [27]. Lipin-1 is also thought to regulate the transcription of genes involved in adipocyte differentiation and fat synthesis and storage [28]. To investigate the antiadipogenic mechanism, the effects of FAVA on mRNA expression levels of PPAR*γ*, C/EBP*α*, and lipin-1 were determined in the epididymal fat pad. The expression of both adipogenic genes, PPAR*γ* and C/EBP*α*, was significantly decreased by FAVA (Figures 4(b) and 4(c)). Additionally, FAVA significantly suppressed lipin-1 expression by 95.6 ± 0.5% compared with that of the HFD group and was greater than that of the orlistat group as a positive control.

### 3.5. Effects of FAVA on Hepatic Histology of HFD-Fed C57BL/6 Mice and mRNA Levels of Cholesterol Biosynthesis in Liver

**Figure 5:**
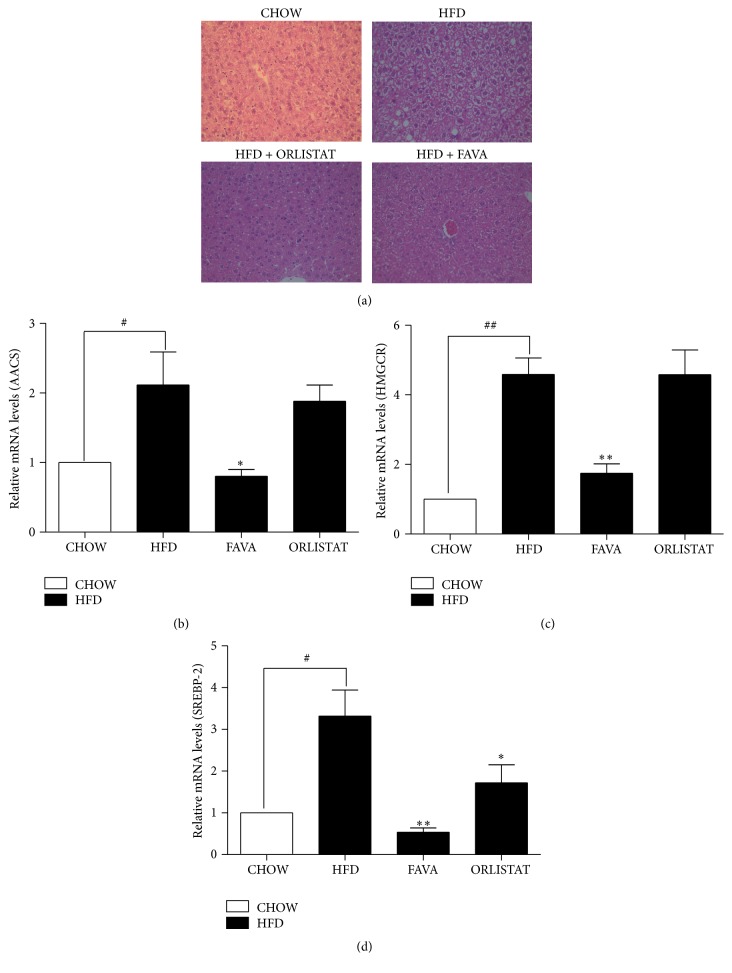
Effect of FAVA on hepatic steatosis and mRNA expressions of cholesterol biosynthesis in the liver of mice. (a) Hematoxylin and eosin staining of liver from mice fed chow diet (CHOW), high-fat diet (HFD), or high-fat diet supplemented with orlistat at 60 mg/kg/day (HFD + ORLISTAT) or high-fat diet supplemented with FAVA at 200 mg/kg/day (HFD + FAVA) (40x magnification). ((b)–(d)) The graphs represent mRNA expression of cholesterol synthesis factors AACS (b), HMGCR (c), and SREBP2 (d), which was analyzed by real-time PCR. The data represent the mean ± SEM (^#^ *p* < 0.05 and ^##^ *p* < 0.005 versus the CHOW group; ^*∗*^ *p* < 0.05 and ^*∗∗*^ *p* < 0.005 versus the HFD group, *n* = 5).

A common characteristic among people with obesity is the development of fatty liver [29, 30]. Therefore, we also analyzed the effect of FAVA on fatty liver development. Histological evaluation is regarded as the “gold standard” for assessing the presence and severity of NAFLD [31]. We histologically evaluated liver sections to determine the extent to which FAVA attenuated hepatic steatosis development. As shown in Figure 5(a), mild fatty liver was observed in mice that were fed a high-fat diet without FAVA. However, a marked reduction in the degree of steatosis was shown in livers from high-fat diet mice treated with FAVA. Moreover, FAVA treatment also decreased total serum cholesterol in mice to 13.7 ± 3.4% (Figure 3(c)). Therefore, we investigated whether SREBP-2, AACS, and HMGCR RNA in the mouse liver were induced by FAVA. Total RNA was prepared from mouse livers, and SREBP-2, AACS, and HMGCR mRNA levels were quantified using real-time PCR. SREBP-2, AACS, and HMGCR mRNA levels were dramatically suppressed in the mice that were fed FAVA (Figures 5(b) and 5(c)).

## 4. Discussion

Our study is the first to demonstrate that FAVA prevents weight gain in HFD-induced obesity in C57Bl/6 mice. Our results showed that body weight gain in groups fed a diet supplemented with FAVA was reduced compared with control HFD mice (Figure 1(a)). Epididymal, mesenteric, and subcutaneous fat pads in C57BL/6 mice were significantly reduced by FAVA supplementation (Figures 1(c)–1(e)). There was a significant reduction in subcutaneous and abdominal fat mass in FAVA-fed groups compared with the HFD group (Figures 2(a)–2(c)). Subcutaneous fat and abdominal fat are the major types of white adipose tissue. Abdominal obesity is associated with an increased risk of cardiovascular diseases and insulin resistance [32]. This study also provides evidence that dietary supplementation of FAVA protects against hepatic steatosis development (Figure 5(a)). We have considered the possibility that the effect of FAVA may be mediated through food intake because decreased food intake would be expected to significantly affect body weight, which influences hepatic steatosis. In this study, however, there was no difference in food intake-induced increase of body weight between the FAVA-fed and non-FAVA-fed groups (Figure 1(b)). This result suggests that FAVA directly protected against obesity and hepatic steatosis independent of food intake.

Obesity is most likely to cause hyperlipidemia, which is considered the leading cardiovascular risk. The hallmark of dyslipidemia in obesity is hypertriglyceridemia in combination with the preponderance of high LDL and low HDL cholesterol [33]. This study shows that, in high-fat diet-fed mice, FAVA supplementation significantly reduced serum levels of cholesterol and the ratio of LDL cholesterol/total cholesterol (Figures 3(c) and 3(d)). Furthermore, insulin levels were increased in the HFD group and were decreased significantly by FAVA supplementation (Figure 3(a)). In the case of prediabetes, increases of blood glucose stimulate the secretion of insulin and subsequently induce hyperinsulinemia to a normal blood glucose range. Hyperinsulinemia, which is a biomarker of insulin resistance, is frequently accompanied by obesity [34]. Leptin is a fat-derived hormone that plays an important role in appetite control and energy expenditure [35]. It has been reported that the concentration of serum leptin is associated with general adiposity and reflects the body fat content [36]. In this report, it was demonstrated that FAVA treatment suppressed the plasma leptin level in mice fed with HFD (Figure 3(b)). Moreover, the weight of adipose tissues strongly correlated with the plasma leptin level. These results confirm that FAVA treatment exerted an antiobesity effect in the diet-induced obesity C57BL/6 mouse model.

PPAR-*γ*, a transcription factor predominantly expressed in adipose tissue, plays an essential role in adipocyte differentiation, lipid storage, and glucose homeostasis [37]. Additionally, adipogenesis is highly regulated by two primary adipogenic transcription factors, PPAR-*γ* and C/EBPs [38]. Among those factors, PPAR-*γ* is well known as the key regulator of adipogenic transcription [10]. PPAR-*γ* is also known to bind to the C/EBP-*α* promoter region that induces the expression of C/EBP-*α* [39]. C/EBP-*α* is a promising candidate transcription factor for directly controlling adipocyte differentiation [40]. We found that FAVA significantly downregulated PPAR-*γ* and C/EBP-*α* mRNA levels in the epididymal fat pad. This effect might be explained in two ways: FAVA either inhibited PPAR-*γ* and C/EBP-*α* or suppressed the upstream molecules. Lipin-1 is also required in adipocyte differentiation for the induction of the adipogenic gene transcription [12]. We found that FAVA could inhibit adipocyte differentiation through the suppression of lipin-1.

Acetoacetyl-CoA synthetase (AACS) can facilitate the incorporation of ketones into lipogenesis [13]. Hasegawa et al. [13] demonstrated that the AACS gene, which encodes the ketone body-utilizing enzyme, is transcriptionally regulated by SREBP-2 and the knockdown of SREBP-2 induced downregulation of AACS and HMGCR gene expression. Additionally, ketone body metabolism via AACS plays an essential role in cholesterol homeostasis. In this study, we showed that the treatment of mice with FAVA resulted in a decrease of SREBP2, AACS, and HMGCR mRNA levels. Therefore, our results suggest that FAVA improves obesity, hyperlipidemia, and NAFLD and that FAVA treatment might be a promising adjuvant therapy in the management of these metabolic disorders.

## 5. Conclusions

FAVA had a marked inhibitory effect on the development of obesity and NAFLD in a high-fat diet-induced obesity mouse model. Inhibiting transcription factors and adipocyte-specific lipogenic genes and decreasing cholesterol synthesis are two possible mechanisms for the antiobesity effect of FAVA. This study suggests that FAVA might be a potential dietary supplement for preventing obesity and NAFLD.

## Figures and Tables

**Table 1:** Primers used in the reverse transcriptase-polymerase chain reaction analysis.

Gene name	Accession number		Sequence
Lipin-1	NM_172950	Forward	5′-TCA GAC ACT TTC AGT AAC TTC AC-3′
		Reverse	5′-TAT CAG CCT TCC CAG CAG-3′

C/EBP-*α*	NM_007678	Forward	5′-CGT CTA AGA TGA GGG AGT C-3′
		Reverse	5′-GGC ACA AGG TTA CTT CCT-3′

PPAR-*γ*	NM_001127330	Forward	5′-GAA AGA CAA CGG ACA AAT CAC-3′
		Reverse	5′-GAA ACT GGC ACC CTT GAA-3′

AACS	NM_030210	Forward	5′-AAG CCC AGA GTT ACG AGT AT-3′
		Reverse	5′-ACA CAG GAA TAG AGG AGT TCT-3′

HMGCR	NM_008255	Forward	5′-AGA ATA ATG TGC TAA GTA GTG CTA A-3′
		Reverse	5′-GCC TCT CTG AAC AAA GAC TC-3′

SREBP-2	NM_033218	Forward	5′-GCG ACC AGG AAG AAG AGA-3′
		Reverse	5′-ACA AAT CCC ACA GAG TCC A-3′

*β*-actin	NM_007393	Forward	5′-GGG AAG GTG ACA GCA TTG-3′
		Reverse	5′-ATG AAG TAT TAA GGC GGA AGA TT-3′

C/EBP-*α*: CCAAT/enhancer-binding protein-*α*; PPAR-*γ*: peroxisome proliferator-activated receptor-*γ*; AACS: acetoacetyl-CoA synthetase; HMGCR: HMG-CoA reductase; and SREBP-2: sterol regulatory element binding protein-2.
